# Interaction Between *Duddingtonia flagrans* and *Pochonia chlamydosporia* for the Biological Control of Bovine Gastrointestinal Nematodes

**DOI:** 10.3390/microorganisms14010085

**Published:** 2025-12-30

**Authors:** Maria Larissa Bitencourt Vidal, Júlia dos Santos Fonseca, Ítalo Stoupa Vieira, Lorena Souza Castro Altoé, Lorendane Millena de Carvalho, Wagner Nunes Rodrigues, Isabella Vilhena Freire Martins, Jackson Victor de Araújo

**Affiliations:** 1 Department of Veterinary, Universidade Federal de Viçosa (UFV), Viçosa 36570-900, MG, Brazil; 2 Department of Veterinary, Centro Universitário UNIFACIG, Manhuaçu 36904-000, MG, Brazil; 3 Department of Epidemiology and Public Health, Federal Rural University of Rio de Janeiro (UFRRJ), Seropédica 23890-000, RJ, Brazil; 4 Department of Veterinary, Faculdade Vértice, UNIVÉRTIX, Matipó 35367-000, MG, Brazil; 5 Department of Veterinary, Universidade do Recôncavo da Bahia (UFRB), Cruz das Almas 44380-000, BA, Brazil; 6 Department of Veterinary and Agronomy, Universidade Federal do Espírito Santo (UFES), Alegre 29500-000, ES, Brazil

**Keywords:** helminths, pasture contamination, sustainable parasite control

## Abstract

Gastrointestinal nematodes are among the most significant parasites affecting livestock health and productivity, leading to major economic losses and contributing to the global increase in resistance to anthelmintics. Biological control using fungi with ovicidal and nematophagous activity offers an environmentally friendly alternative. This trial represents the first long-term field evaluation in cattle of the commercial combination of *D. flagrans* and *P. chlamydosporia* under natural infection conditions. Eighteen Holstein × Zebu males (12–15 months old) were divided into three groups (n = 6): T1 (*D. flagrans*), T2 (*D. flagrans + P. chlamydosporia*), and control. Treatments were administered orally daily (6 g/100 kg BW of each fungus; 10^6^ chlamydospores/g) for nine months. Fecal egg counts (EPG) and infective larvae in pasture (L3) were monitored. Groups T1 and T2 showed significantly lower EPG values than the control during most of the experimental period. *Haemonchus* spp. was identified as the predominant nematode, supporting its epidemiological relevance. The combined fungal treatment exhibited enhanced effectiveness, enhancing parasite suppression through complementary ovicidal and larvicidal mechanisms. This approach offers a sustainable alternative to the excessive use of chemical compounds and has the potential to contribute to integrated animal health and livestock production.

## 1. Introduction

Brazil’s commercial dairy herd is of worldwide importance and the state of Minas Gerais, in the southeastern region of Brazil, is particularly noteworthy for its dairy farming. Within the contemporary scenario of ruminant farming, there is a growing emphasis on enhancing production levels while simultaneously ensuring the wellbeing of animals. Gastrointestinal nematode (GIN) control plays a crucial role in addressing both of these essential considerations [1].

Animals become infected with GINs mainly through exposure to pastures contaminated with third-stage larvae (L3) [2]. The main way to control these parasites is through treating animals with anthelmintic drugs [3,4] but in many cases these are administered incorrectly, with excessive and indiscriminate use of therapeutic agents. This increases production costs without achieving effective control of infections [5]. In addition, anthelmintics do not act on the free-living stages of nematodes and can leave harmful residues in animal products [6,7,8].

Protocols for eliminating GINs, such as those of the genera *Haemonchus* and *Cooperia*, consist of use of drug therapies based on synthetic chemical compounds. However, a wide variety of studies have demonstrated helminth resistance to commercially available drugs [3,4,5]. Control with bioproducts is an alternative for managing cattle nematodes in pastures [7]. The use of biological control methods involving nematophagous fungi has proven to be a safe and viable alternative [7,9,10,11,12] that complements GIN control activities through the action of these fungi in the environment.

Promising results have been obtained with various species of nematophagous fungi, in particular *Duddingtonia flagrans* for controlling helminth larvae and *Pochonia chlamydosporia* with ovicidal action towards controlling helminth eggs [11,13,14,15,16,17,18,19].

The use of multiple fungal species has been increasingly highlighted in the literature, as it enables the combination of distinct mechanisms of action for the control of helminths. Effective associative use typically requires pairing an ovicidal species with a larvicidal one, such as the interaction between *D. flagrans* and *P. chlamydosporia*. Predatory fungi including *D. flagrans*, *Arthrobotrys* spp., and *Monacrosporium* spp. are employed to target infective larval stages in pastures, forming trap-like structures capable of capturing and destroying infective larvae. In contrast, ovicidal fungi such as *P. chlamydosporia*, *Mucor circinelloides*, and *Paecilomyces lilacinus* do not form trapping networks; instead, they penetrate eggs through a mechanical enzymatic process, subsequently colonizing the internal contents and rendering the eggs unviable [17].

The ovicidal activity of *P. chlamydosporia*, both as a single agent and in association with other fungi, has been demonstrated against eggs from various hosts, under both in vitro and field conditions. Isolates VC1 and VC4 have been widely tested in vitro against eggs of different helminth species, consistently showing high ovicidal efficacy and strong potential for formulation and commercial application. This species is particularly effective against helminths that persist in the environment primarily in the egg stage [17,20]. However, Fonseca et al. (2022) [20] reported limited effects of a formulation containing only *P. chlamydosporia* in cattle, suggesting that the short duration of the egg stage in pastures may have reduced the opportunity for fungal action. In this context, the association of fungal species with complementary mechanisms of action has shown clear advantages [21].

The literature indicates that combining two or more fungal species can result in enhanced effectiveness, substantially increasing efficacy without evidence of antagonism between isolates. For example, Carmo et al. (2023) [21] reported successful control of equine gastrointestinal nematodes in field conditions using a combined formulation of *D. flagrans* and *P. chlamydosporia* [22].

Currently, commercial formulations containing *D. flagrans* are available, such as BioWorma^®^ (Australia) and BioVerm^®^ (Brazil), both designed to control infective larval stages of gastrointestinal nematodes in grazing animals. The development of biological control strategies relies on selecting highly effective fungal isolates, and decades of research have demonstrated the strong individual performance of several species [23]. Although isolated species may exhibit excellent efficacy, their association represents an even more promising approach, integrating complementary actions and broadening the potential of future formulations.

However, to date, no long-term field studies have evaluated the combined application of these two fungi in cattle naturally infected with gastrointestinal nematodes. This gap limits our understanding of their potential complementary effectiveness under real production conditions.

Therefore, the aim of this study was to evaluate the effects of two commercial bioproducts, one based on *D. flagrans* (Bioverm^®^) and the other consisting of an association between *D. flagrans* and *P. chlamydosporia,* on reducing the levels of nematode eggs in animal feces and larval infection in pastures.

## 2. Materials and Methods

Two formulations supplied by GhenVet Saúde Animal (Brazil) were used: a formulation based on the fungus *D. flagrans* (isolate AC001) known as Bioverm^®^, which contains 10^6^ chlamydospores of the fungus per gram, and an experimental formulation (EF) combining *D. flagrans* (isolate AC001) and *P. chlamydosporia* (isolate VC04), containing 10^6^ chlamydospores of each fungus per gram. Individual or combined administration was carried out at a dosage of 6 g/100 kg live weight.

The viability of *P. chlamydosporia* chlamydospores was verified prior to beginning of the experiment by the company responsible for supplying the material, following its internal quality control procedures. The formulations were carefully stored under controlled temperature conditions and protected from light to preserve stability. In addition, previous studies have demonstrated that *P. chlamydosporia* is capable of surviving passage through the gastrointestinal tract of ruminants and is recovered from feces in a viable form [24,25].

The experiment was conducted on a farm located in the municipality of Abre Campo, state of Minas Gerais, southeastern Brazil, latitude 20°18′04″ S, longitude 42°28′39″ W, from February to October 2021. The region presents a Cwa climate according to the Köppen–Geiger classification, characterized by a well-defined rainy season, high annual humidity, and mild temperatures typical of tropical highland environments, which provides relatively stable environmental conditions for L3 survival and migration.

Initially, for monitoring, feces from 24 animals were collected and processed to determine eggs per gram of feces (EPG) counts on a weekly basis, in accordance with the methods of Gordon and Whitlock [23] and modifications of Dennis, Stone and Swanson [26]; while pasture forage samples were analyzed on a fortnightly basis, in accordance with the technique described by Raynaud and Gruner [27]. After the initial test, the animals were monitored fortnightly throughout the experiment using the same techniques described above. The infective larvae (L3) were identified in accordance with Keith [28].

Based on this analysis, eighteen male Holstein-Zebu crossbred cattle, aged between 12 and 15 months, with an average initial weight of 150 kg, which remained at this average weight throughout the experiment, were pre-treated with an anthelmintic suspension of 15% albendazole sulfoxide (Agebendazol^®^) by injection, in a single dose of 1 mL/44 kg of body weight.

Prior to the experiment, all animals received a single dose of a commercial injectable anthelmintic containing albendazole sulfoxide, administered subcutaneously according to the manufacturer’s recommendations, to standardize parasite burdens before group allocation. After a washout period, animals were ranked by individual fecal egg counts and allocated to the Control, Bioverm, or Association groups using blocked randomization to ensure similar baseline EPG among groups. All groups were managed under identical pasture, handling, and husbandry conditions, differing only in the antiparasitic treatment applied. Fecal egg counts and pasture larval recovery were performed using standardized laboratory methods, with assessors blinded to group allocation.

Twenty-one days after anthelmintic treatment, and after supporting the absence of nematode eggs in the feces using the EPG technique of Gordon and Whitlock [23], a field test was carried out using fungus treatments on naturally infected animals, as described in Ordinance 88 of the Ministry of Agriculture, Livestock and Supply [29].

The animals were divided according to their average EPG, into three groups of six animals each and separated into three 6-ha paddocks of *Brachiaria brizantha*, naturally infected with nematode larvae as a result of previous grazing by animals naturally infected with GINs. Although specific microenvironmental variables (shade, humidity, sward height) were not recorded, the Cwa climate of Abre Campo (MG), with high annual humidity, provides conditions suitable for L3 survival and migration.

Each treatment group was allocated to a single 6-ha paddock throughout the experimental period. Therefore, the paddock was confounded with treatment and did not constitute an independent experimental replicate. To mitigate potential bias from this design, paddocks were selected based on their high homogeneity regarding soil type, forage species (*B. brizantha*), and identical previous grazing history.

In the group that received Bioverm^®^ (T1), each animal was treated with 6 g of the product for every 100 kg of body weight. The product was administered daily, mixed with 1 kg of maize bran. Group T2 received the experimental formulation (mixed with maize meal), also at a daily dose of 6 g/100 kg body weight. In the control group (C), each animal received maize meal daily, without fungal spores. The animals were monitored fortnightly and the dosage of the products was based on body score and average weight, in accordance with previous studies carried out on the same farm, with the same animals, as described by Vieira et al. [13] and Oliveira et al. [14].

Every 15 days from the start of the experiment, two samples of *B. brizantha* grass were collected (0–20 cm and 20–40 cm away from the feces) in the grazing areas of the treated and control groups, at six different points, as described by Raynaud and Gruner [27,30]. Samples of 500 g of pasture forage were collected and processed for recovery of infective larvae (L3) using a modified Baermann technique, following standard procedures for pasture larval migration and recovery. The sediment was examined under an optical microscope and the larvae were counted and identified using the criteria established by Keith [28]. The 500 g samples of grass used were placed in an oven at 100 °C to obtain the dry matter. The data obtained were transformed into the number of larvae per kilogram of dry matter.

The experiment lasted nine months (February to October 2021), during which time animal feces and pasture vegetation were collected fortnightly. The study was also approved by the Ethics Committee for the Use of Animals (CEUA) of the Federal University of Viçosa (UFV), under reference number 37/2020.

Climate data on minimum, average and maximum monthly temperatures and monthly precipitation were obtained from the Agricultural Meteorological Monitoring System (Agritempo), available at the website https://www.agritempo.gov.br/br/estado/MG/monitoramento/ (accessed on 26 December 2012). The averages of EPG, L3 recovered from coprocultures, and L3 obtained from pasture samples, as well as the climatic data collected throughout the nine months of the experiment, were organised into monthly values.

### Statistical Analyses

A split-plot ANOVA was applied, with treatment (Control, T1, and T2) as the main-plot fixed factor and time (monthly measurements) as the subplot factor. Animals nested within treatment were considered the experimental units for animal-level outcomes (EPG), and the treatment × time interaction was tested. Pasture L3 data were interpreted descriptively at the paddock level due to the lack of independent paddock replication.

Subsequently, the EPG, larval migration and body condition score datasets were analysed using analysis of variance (ANOVA) in a split-plot design over time, with parasite managements as main plots and evaluation periods as subplots. When significant effects were detected, Tukey’s test at 5% probability was applied for comparison of means. In addition, treatment efficacy was assessed using the Fecal Egg Count Reduction Test (FECRT). All statistical analyses were performed using SISVAR 5.6.

Although generalized linear mixed models are recommended for overdispersed count data, exploratory attempts indicated model instability due to sample size and zero inflation; therefore, a simplified descriptive approach was retained.

EPG and pasture L3 counts are count data and are known to present overdispersion and repeated measures over time. In this long-term field trial, statistical analyses were performed using arithmetic monthly means and a split-plot ANOVA as a descriptive approach to compare overall treatment effects across the experimental period. Given the limited number of animals per group and the complexity of the experimental design, more complex generalized linear mixed models were not applied. No transformation was applied to EPG data, following WAAVP recommendations for field studies.

## 3. Results

### 3.1. Body Condition Score (BCS)

**Figure 1:**
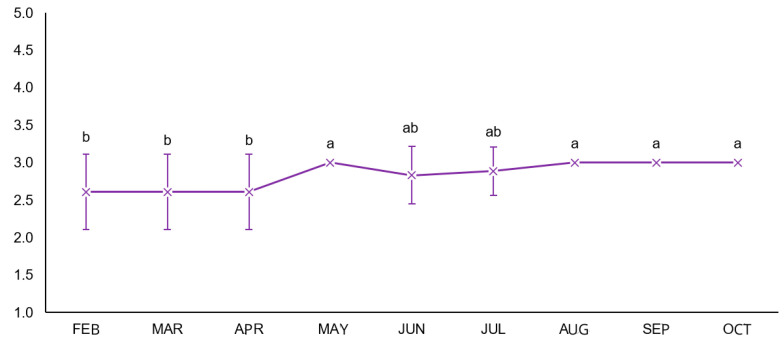
Body condition score (BCS) dynamics over time (means followed by the same letter among parasite treatments do not differ statistically from each other, according to Tukey’s test, at 5% probability).

The body condition score results are shown in Figure 1. In the split-plot ANOVA, no interaction was observed between time and treatment (mean square = 0.025), and no significant differences occurred among the parasite management strategies (mean square = 0.025). Time was the only significant factor (mean square = 0.589), indicating that BCS increased progressively during the evaluation period, regardless of treatment.

### 3.2. Egg Counting Techniques

**Figure 2:**
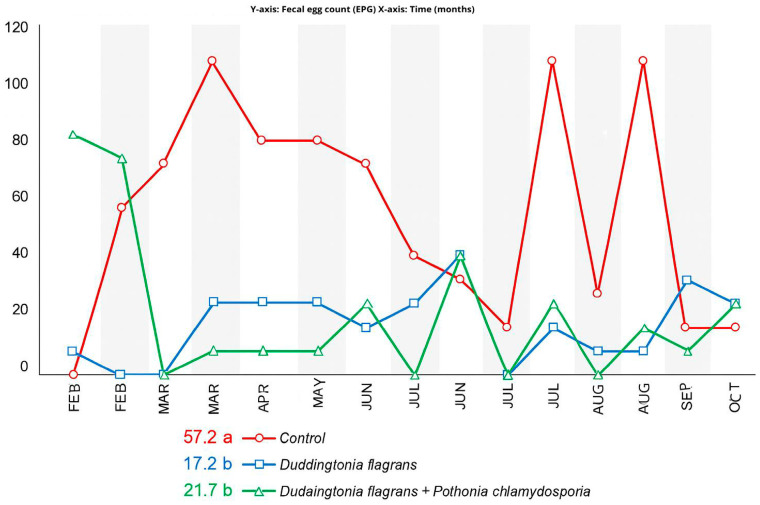
Dynamics of egg count per gram of feces (EPG) over time as a function of parasite management (means followed by the same letter among parasite management methods do not differ statistically from each other, according to Tukey’s test, at a 5% probability).

The monthly fecal egg count (EPG) values for the three experimental groups are presented in Figure 2. According to the ANOVA performed under a split-plot design over time, a significant effect of parasite management was detected (*p* < 0.01), whereas no interaction between treatment and sampling period was observed.

During the first month of evaluation (February), no statistical differences in EPG were found among the groups. In March, animals receiving Treatment 1 (*Duddingtonia flagrans*, Bioverm^®^) exhibited significantly lower EPG values than the control (Tukey, *p* < 0.05). Across the experimental period, both Treatment 1 and Treatment 2 showed lower mean EPG values than the control, particularly during months with more favorable climatic conditions for parasite development. From June onwards, including August, September and October, EPG values no longer differed statistically among treatments, which coincided with lower climatic favourability for larval development.

**Figure 3:**
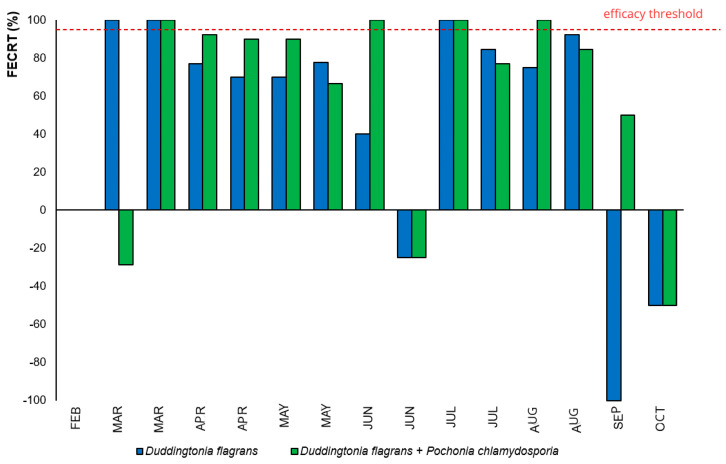
Testing for reduction in fecal egg count (FECRT) over time as a function of biological parasite management.

Nevertheless, when evaluated through the Fecal Egg Count Reduction Test (FECRT) (Figure 3), the fungal association (Treatment 2) displayed >95% efficacy during a greater number of months compared to *D. flagrans* alone, evidencing enhanced and prolonged suppression of egg shedding due to the enhanced effectiveness of both fungi.

### 3.3. Larval Recovery Techniques

**Figure 4:**
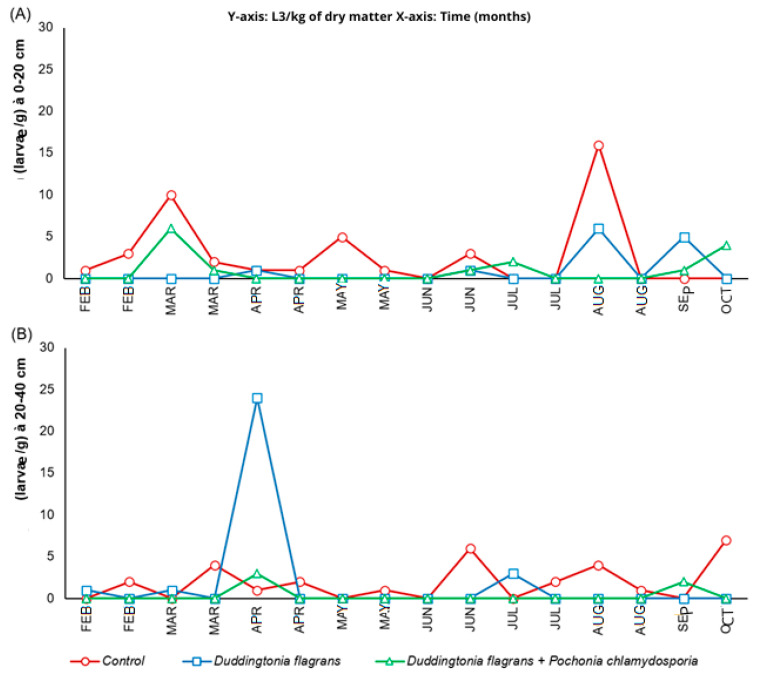
Active migration dynamics (Baermann method) over time for distances of 0–20 cm (**A**) and 20–40 cm (**B**) as a function of parasite management (L3/kg of dry matter).

The quantities of infective L3 larvae obtained from coprocultures are shown in Figure 4. In Treatment 2 and the control group, *Haemonchus* sp. was the predominant genus, followed by *Cooperia* sp., throughout most of the experimental period. Notably, after the beginning of the trial, L3 larvae of both genera were no longer detected in Treatment 1 (Bioverm^®^), indicating a strong larvicidal activity of *D. flagrans* in fecal material.

According to ANOVA (split-plot design), significant differences among treatments were observed for L3 recovery at both distances evaluated (*p* ≤ 0.05). These differences should be interpreted with caution, as pasture measurements were obtained from single paddocks per treatment and therefore represent group-level trends rather than replicated pasture-level effects. At 0–20 cm from the fecal mass, Treatment 1 was associated with a 69% reduction in larval migration, while the fungal association (Treatment 2) produced a 65% reduction compared to the control. At the greater distance (20–40 cm), Treatment 2 showed a lower larval recovery, reaching an 83% reduction, in contrast to the minimal reduction recorded for Treatment 1 (3%). This finding suggests the importance of incorporating an ovicidal fungus (*P. chlamydosporia*) to limit larval recruitment into the environment, potentially enhancing the environmental protective effect relative to *D. flagrans* alone under the conditions of this study.

Although T1 showed a greater reduction in L3 in coprocultures, field data indicated that T2 reduced L3 availability more effectively at 20–40 cm from fecal pats. This difference likely reflects the contrast between controlled coproculture conditions and the complex pasture environment, where factors such as humidity, vegetation structure, and soil–feces interactions influence L3 migration and persistence.

### 3.4. Environmental Conditions

**Figure 5:**
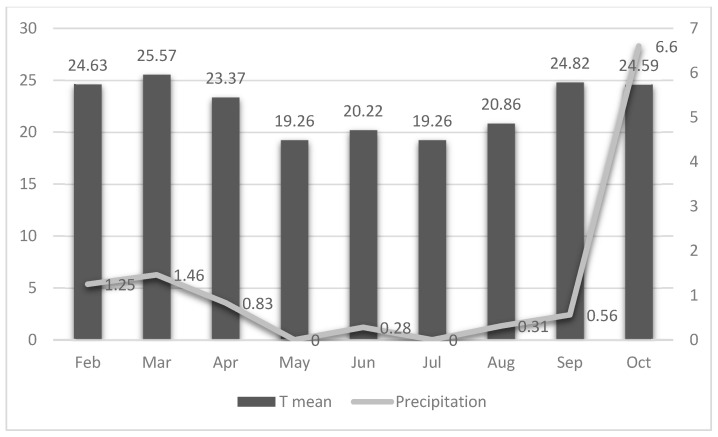
Mean monthly temperatures (T mean) and precipitation (mm) from February to October 2021 in the Abre Campo region, Minas Gerais, Brazil obtained from the Agritempo meteorological station closest to the experimental area.

As observed in coprocultures, the larvae recovered from pastures in the early months belonged predominantly to the genera *Haemonchus* and *Cooperia*. These patterns aligned closely with the climatic conditions recorded during the study period. Monthly minimum, average, and maximum temperatures, along with precipitation data, are shown in Figure 5.

The lowest temperatures occurred in May and July, with monthly averages ranging from 19.26 °C (July) to 25.57 °C (March). Precipitation was highest in October (6.60 mm), while the remaining months exhibited low rainfall, ranging from 0 mm (July) to 1.46 mm (March).

These climatic variables likely influenced the dynamics of larval development and dispersion in the pasture throughout the experiment.

## 4. Discussion

The progressive increase in body condition score (BCS) observed throughout the study (Figure 1), regardless of treatment, suggests that cattle maintained adequate nutritional status during the experimental period. The overall improvement in BCS aligns with previous observations on the same farm, where fungal biological control contributed indirectly to maintaining animal performance and weight gain by reducing parasitic pressure [11,12,13]. These authors reported that even moderate reductions in environmental larval availability can help sustain productive indicators such as body weight and condition in grazing cattle consistent with the pattern observed in the present study, where fungal treatments supported a low parasitic challenge that allowed normal physiological recovery over time. Although both fungi have been previously assessed individually and certain combinations have been tested in other host species, this study represents the first long-term field evaluation in cattle using this specific commercial formulation of *D. flagrans* associated with *P. chlamydosporia*.

Rather than reflecting only direct ovicidal or larvicidal action, the differences observed among treatments must also be interpreted in light of the environmental constraints governing L3 dynamics. Factors such as humidity fluctuations, soil–feces interactions, and microclimatic variation during the unusually dry period may have modulated fungal activity differently across pasture distances. These elements help explain the divergence between coproculture results where controlled conditions favored initial fungal activity and the field observations, where ecological persistence and competitiveness determined the magnitude of L3 reduction.

Epidemiological surveys by Kenyon et al. [31] and Franco et al. [32] indicate that gastrointestinal nematodes are present in approximately 95% of pastures, a finding consistent with the results of the present study. *Haemonchus* and *Cooperia* were the predominant genera identified in coprocultures, in agreement with previous studies conducted in Brazil [11,12,13,15]. These nematodes remain among the most pathogenic for cattle, given their well-established association with anemia, reduced nutrient absorption and mortality [33,34].

The initial decline in fecal egg counts (EPG) across all groups can be attributed to the anthelmintic protocol applied before the trial, as such drugs effectively remove adult parasites and their eggs [4]. Once cattle returned to pasture without further chemical intervention, reinfection occurred, especially in the control group (Figure 2 and Figure 3). Nonetheless, EPG values remained low in all treatments, likely reflecting host age, immunological resistance and reduced environmental larval availability conditions that provided a suitable baseline for evaluating fungal biocontrol.

The experimental design followed MAPA guidelines [29], which establish a controlled framework for assessing antiparasitic interventions in Brazil. However, because no regulatory standards exist for biological control products, studies such as this are essential to support their application in sustainable parasite management.

Helminthophagous fungi have been repeatedly validated as efficient biological control tools [16,17,18,19,21]. Their oral administration is advantageous because fungal propagules reach the fecal mass the environment in which nematode eggs hatch and larvae develop allowing the fungi to either disrupt egg embryonation or trap and kill larvae [35,36]. In this study, both *D. flagrans* alone and its combination with *P. chlamydosporia* reduced EPG compared with the control during specific evaluation months, and both treatments markedly decreased L3 counts on pastures (Figure 4). These results are in line with previous trials conducted on the same farm using other fungal formulations [11,12,15]. Moreover, the combined activity of *D. flagrans* and *P. chlamydosporia* has also been demonstrated in horses, where significant reductions in infective small strongyle larvae were observed [21].

The low environmental contamination observed throughout the study mirrors earlier findings obtained using *A. cladodes* and *P. chlamydosporia* in alginate pellets, which exhibited residual effects in pastures and markedly reduced egg deposition [14]. The consistency among studies reinforces that fungal control depends on the complementary modes of action of each species and the developmental stage they target.

Effective gastrointestinal nematode control requires simultaneous interruption of both the parasitic (host) and free-living (environmental) phases [35,37]. Here, reductions in both EPG and pasture L3 counts support fungal activity in the free-living stage, which is essential for reducing reinfection pressure in grazing systems [12];. Lower environmental larval availability is particularly valuable because it creates a long-term reduction in parasite cycling.

The success of *D. flagrans* in this study is consistent with its biological characteristics. This species forms chlamydospores capable of withstanding gastrointestinal transit and being dispersed through feces [14,35,38]. Once released into feces, *D. flagrans* forms trapping structures that ensnare and destroy newly hatched larvae explaining the absence of L3 in coprocultures from animals treated with this fungus [36,39].

*P. chlamydosporia*, in turn, is an established ovicidal species [40]. Through enzymatic degradation and hyphal penetration, it disrupts helminth egg embryogenesis [19,41]. The reduction in EPG observed in the combination treatment highlights the complementarity between the ovicidal mechanisms of *P. chlamydosporia* and the larvicidal action of *D. flagrans*, evidencing an enhanced effectiveness. This enhanced effectiveness likely contributed to sustained reductions in pasture contamination, even under fluctuating climatic conditions.

Environmental factors also shaped parasite dynamics. Pasture height ranged from 15 to 80 cm, influencing microclimatic conditions that directly affect larval survival and fecal degradation [41,42]. Temperature and precipitation patterns play a central role in larval development [43]. Optimal temperatures for *Haemonchus* and *Cooperia* larvae (13–26 °C) [34] were exceeded during six of the nine months of assessment (Figure 5), and rainfall remained low during autumn and winter, conditions that likely reduced overall larval viability across treatments. Because water availability is essential for L3 migration from feces to forage [30,43] the observed climatic pattern contributed to a general decline in pasture infectivity. Even so, significant differences between treated and untreated groups support that the fungi alone or combined acted consistently to suppress environmental larval loads.

From a practical standpoint, the use of fungal biocontrol in cattle systems is feasible, as formulations can be incorporated into mineral supplements or protein-energy blocks without requiring changes in routine management. The approach has a relatively low operational cost compared with chemical anthelmintics and may offer particular advantages in extensive grazing systems, where strategic treatments are difficult to implement and environmental contamination is high. By reducing pasture infectivity and supporting animal performance, sustained fungal administration could help mitigate the impact of anthelmintic resistance. Nevertheless, further cost–benefit evaluations and long-term assessments are needed to define adoption thresholds under commercial conditions.

We acknowledge that the number of animals per group was limited (n = 6), which may reduce the statistical power and the ability to detect subtle differences among treatments. Nevertheless, the consistent patterns observed across the evaluated parameters support the biological relevance of the findings. Future studies with larger sample sizes are warranted to confirm these results under diverse field conditions.

Given that extensive grazing predominates in Brazilian ruminant production, fungal bioproducts represent a valuable component of integrated parasite management. By reducing pasture contamination and reinfection pressure, they mitigate productivity losses associated with helminthiasis and help decrease reliance on chemical anthelmintics, supporting more sustainable livestock systems.

### Limitations

This study presents some limitations that should be acknowledged. First, the number of animals per group was small (n = 6), which reduces statistical power and may limit the detection of subtle treatment effects. Second, the experiment was conducted in a single geographic location (Abre Campo, MG), and environmental conditions typical of this site including an unusually low rainfall period during the experimental months may influence L3 survival and migration. Third, although fungal viability was assessed prior to administration, continuous monitoring of formulation stability and recovery of *P. chlamydosporia* in feces was not performed, preventing a direct evaluation of gastrointestinal passage and environmental dissemination.

An important limitation of this study is the lack of true replication at the paddock level, as each treatment was applied to a single paddock. This design confounds paddock and treatment effects and limits causal inference regarding pasture-level outcomes, particularly L3 recovery. Consequently, pasture data should be interpreted as descriptive and supportive of animal-level findings, rather than as definitive evidence of treatment effects on pasture contamination. The primary inference of this study therefore relies on animal-level parasitological outcomes measured repeatedly within each group. Future studies should incorporate larger sample sizes, multiple production environments, and systematic monitoring of fungal viability under field conditions.

## 5. Conclusions

In this study, the use of the nematophagous fungus *D. flagrans* (Bioverm^®^), either alone or combined with *P. chlamydosporia*, effectively reduced fecal egg counts and pasture larval contamination. While both treatments contributed to lowering parasite burden, the combined formulation demonstrated a markedly enhanced effect, as it integrates two complementary modes of action: larvicidal activity from *D. flagrans* and ovicidal activity from *P. chlamydosporia*. This enhanced effectiveness resulted in broader and more consistent suppression of gastrointestinal nematodes. Therefore, fungal associations represent a promising and sustainable strategy for integrated helminth control in ruminant production systems, suggesting broader biological effectiveness than single-fungus approaches under the conditions evaluated.

## Data Availability

The original contributions presented in this study are included in the article/Supplementary Materials. Further inquiries can be directed to the corresponding author.

## Supplementary Materials

The following supporting information can be downloaded at: https://www.mdpi.com/article/10.3390/microorganisms14010085/s1, Table S1: Individual animal EPG values over time; Table S2: Pasture L3 counts by sampling distance and date; Table S3: Climatic data used in Figure 5.
