# Supplementary material for: Interaction Between Duddingtonia flagrans and Pochonia chlamydosporia for the Biological Control of Bovine Gastrointestinal Nematodes
Source: Microorganisms. 2025 Dec 30;14(1):85. doi: 10.3390/microorganisms14010085 (PMC12843830; doi:10.3390/microorganisms14010085)
Supplement: Supplementary file 1 [file microorganisms-14-00085-s001.zip › microorganisms-4043837-supplementary.pdf]

Supplementary Materials: Table S1: Individual animal EPG values over time, including group means, variability measures, and sample sizes. Table S2: Pasture L3 counts by sampling distance and date, expressed as L3 per kg of dry matter. Table S3: Climatic data used for Figure 5 with corresponding units.

Table S1. Individual animal EPG values over time

Table S1. Individual fecal egg counts (EPG) of cattle over the experimental period. Values are presented per animal, with group means ± SD and sample size (n) per time point.

February

Animal	EPG
C1	0
C2	0
C3	0
C4	0
C5	0
C6	0

Mean ± SD: 0 ± 0 **n = 6**

March

Animal	EPG
C1	250
C2	0
C3	0
C4	0
C5	0
C6	100

Mean ± SD: 58.3 ± 96.9 **n = 6**

April

Animal	EPG
C1	0
C2	300

C3	0
C4	200
C5	100
C6	50

Mean ± SD: 108.3 ± 113.4 **n = 6**

May

**Animal**	**EPG**
C1	100
C2	200
C3	0
C4	150
C5	0
C6	50

Mean ± SD: 83.3 ± 79.6 **n = 6**

June

**Animal**	**EPG**
C1	50
C2	50
C3	100
C4	50
C5	0
C6	0

Mean ± SD: 41.7 ± 36.4 **n = 6**

July

**Animal**	**EPG**
C1	50
C2	0

C3	0
C4	0
C5	50
C6	0

Mean ± SD: 16.7 ± 25.8 **n = 6**

August

**Animal**	**EPG**
C1	50
C2	50
C3	50
C4	0
C5	50
C6	0

Mean ± SD: 33.3 ± 25.8 **n = 6**

September

**Animal**	**EPG**
C1	50
C2	50
C3	0
C4	0
C5	0
C6	0

Mean ± SD: 16.7 ± 25.8 **n = 6**

October

**Animal**	**EPG**
C1	0
C2	0
C3	0

C4	100
C5	0
C6	0

Mean ± SD: 16.7 ± 40.8 **n = 6**

Table S2. Pasture L3 counts by sampling distance and date

Table S2. Recovery of infective larvae (L3) from pasture at different distances from fecal pats, expressed as L3 per kg of dry matter.

Control

**Month**	**Distance**	**Mean L3/kg DM**
Feb	0–20 cm	2.0
Feb	20–40 cm	1.0
Mar	0–20 cm	6.0
Mar	20–40 cm	2.0
Apr	0–20 cm	1.0
Apr	20–40 cm	1.5
May	0–20 cm	3.0
May	20–40 cm	0.5
Jun	0–20 cm	1.5
Jun	20–40 cm	3.0
Jul	0–20 cm	0.0
Jul	20–40 cm	1.0
Aug	0–20 cm	8.0
Aug	20–40 cm	2.5
Sep	0–20 cm	0.0
Sep	20–40 cm	0.0
Oct	0–20 cm	0.0
Oct	20–40 cm	7.0

Bioverm®

Month	Distance	Mean L3/kg DM
Feb	0–20 cm	0.0
Feb	20–40 cm	0.5
Mar	0–20 cm	0.0
Mar	20–40 cm	0.5
Apr	0–20 cm	0.5
Apr	20–40 cm	12.0
May	0–20 cm	0.0
May	20–40 cm	0.0
Jun	0–20 cm	0.5
Jun	20–40 cm	0.0
Jul	0–20 cm	0.0
Jul	20–40 cm	1.5
Aug	0–20 cm	3.0
Aug	20–40 cm	0.0
Sep	0–20 cm	5.0
Sep	20–40 cm	0.0
Oct	0–20 cm	0.0
Oct	20–40 cm	0.0

Association (*D. flagrans* + *P. chlamydosporia*)

Month	Distance	Mean L3/kg DM
Feb	0–20 cm	0.0
Feb	20–40 cm	0.0
Mar	0–20 cm	3.5
Mar	20–40 cm	0.0
Apr	0–20 cm	0.0
Apr	20–40 cm	1.5
May	0–20 cm	0.0

May	20–40 cm	0.0
Jun	0–20 cm	0.5
Jun	20–40 cm	0.0
Jul	0–20 cm	1.0
Jul	20–40 cm	0.0
Aug	0–20 cm	0.0
Aug	20–40 cm	0.0
Sep	0–20 cm	1.0
Sep	20–40 cm	1.0
Oct	0–20 cm	4.0
Oct	20–40 cm	0.0

Table S3. Climatic data used in Figure 5

Table S3. Monthly climatic data recorded during the experimental period and used for Figure 5.

Month/Year	Tmin (°C)	Tmean (°C)	Tmax (°C)	Precipitation (mm)
Feb 2021	21.3	25.5	30.5	237.3
Mar 2021	20.0	25.6	31.5	46.6
Apr 2021	17.6	23.4	29.3	25.7
May 2021	15.7	22.3	28.5	6.1
Jun 2021	14.5	20.5	26.6	8.4
Jul 2021	12.4	19.4	26.9	0.0
Aug 2021	14.1	20.8	27.8	10.8
Sep 2021	17.9	24.9	33.5	11.9
Oct 2021	20.2	24.9	30.6	168.4
